# Editorial: Mortality of people with severe mental illness: Causes and ways of its reduction

**DOI:** 10.3389/fpsyt.2022.1009772

**Published:** 2022-08-18

**Authors:** Mario Luciano, Maurizio Pompili, Norman Sartorius, Andrea Fiorillo

**Affiliations:** ^1^Department of Psychiatry, University of Campania “Luigi Vanvitelli”, Naples, Italy; ^2^Department of Neurosciences, Mental Health, and Sensory Organs, Faculty of Medicine and Psychology, Suicide Prevention Centre, Sant'Andrea Hospital, Sapienza University of Rome, Rome, Italy; ^3^Association for the Improvement of Mental Health Programmes (AMH), Geneva, Switzerland

**Keywords:** lifestyle, mental disorders, physical activity, mortality, morbidity

Severe mental disorders (SMD) are associated with a variety of other illnesses and have poorer health outcomes and higher mortality than other non-communicable disease (1–3). People with severe mental disorders die on average 10–20 years earlier than the general population (4); this gap is increasing over time and recent data suggest that their standardized mortality ratios are higher than those previously reported (5). Only a minority of deaths of people with SMD are attributable to unnatural causes, such as suicide, homicide or accidents (6, 7): the majority of deaths are due to physical disorders, such as cardiovascular, respiratory and infectious diseases, diabetes mellitus and cancers (8, 9).

During the COVID-19 pandemic the mortality rates of patients with SMIs were even higher, due to physical complications of COVID-19 infection (10) and reduced access to care. Compared with the general population, people with SMI have a significantly higher risk of being infected by COVID-19, and of being hospitalized in intensive care units due to COVID-19 complications (11). This increased risk is due to several factors, including: (1) the presence of cognitive dysfunctions in people with SMI, which diminish compliance with preventive behavior (12); (2) the higher prevalence of comorbid medical conditions that are associated with severe forms of COVID-19 illness; (3) socioeconomic disadvantages, which result in unsafe working and living environments. Moreover, available data on COVID-19 infection suggest that people with severe mental disorders are more likely to be infected and die because of COVID-19 sequelae (13, 14). For these reasons, it has been advocated that patients with SMD should be given priority in programs of vaccination against COVID-19 (11, 15).

The increased morbidity and mortality in patients with severe mental disorders is also due to unhealthy lifestyle behaviors. Compared with the general population, patients with SDM have higher rates of sedentary behaviors, of tobacco smoking and of unhealthy diet (16, 17). Moreover, these patients are less likely to comply with appropriate interventions to correct unhealthy lifestyle behaviors and to seek medical help for physical diseases (18).

Among other factors that can contribute to the excess of morbidity and mortality in patients with severe mental disorders, various forms of stigma play an important role. Stigma leads to professionals' negative attitudes toward people with mental disorders, and to discrimination in the process of health care. Side-effects of many psychotropic medications and drugs are also contributing to the increased vulnerability to physical illness of people with SMD (19, 20).

More recently, the increased comorbidity between SMDs and physical disorders was seen as being related to common etiopathogenetic factors of SMD and other disorders, including the involvement of the immunological system, inflammation, and mitochondrial dysfunction (21). In fact, a low-grade systemic inflammatory state has been reported both in patients with SMD and in patients with metabolic syndrome, type 2 diabetes mellitus, moderate to severe obesity and hypertension (22).

Moreover, this evidence is further supported by the fact that interventions aimed at improving physical health activities or at rebalancing unhealthy as well as attention to diet habits in people with severe mental disorders are associated with a reduction of inflammatory state, through a reduction in blood levels of BDNF and pro-inflammatory cytokines, which leads to an improvement of health and a recovery from both physical and mental illnesses (23, 24).

From a public health perspective, the comorbidity between mental and physical disorders should be now considered a major health problem. Parallel to the public awareness of the magnitude of this problem, it became clear that a single discipline approach will not be able to identify effective solutions and that a multilevel approach, including the involvement of different health professionals and, stakeholders, patients and relatives, is needed for a proper long-term management of physical and mental health conditions.

The importance of lifestyle for the maintenance of physical and mental health led to the development of several psychosocial and behavioral interventions aiming at an improvement patients' physical health and dietary patterns and a reduction of alcohol abuse and tobacco smoking. The ultimate aims of these interventions were the reduction of cardiovascular risk factors and of the Body Mass Index (BMI). In addition to encouraging results, these studies also made it clear that that the improvement of physical health of people with severe mental disorders also leads to an improvement of mental health and progress in several other health-related domains, such as patients' empowerment, improved social contacts, and a reduction of the numbers of relapses and hospitalizations (16).

The focus of the Research Topic “*Mortality of People with Severe Mental Illness: Causes and Ways of its Reduction*” is to improve the understanding of the complex relationship between the higher rates of mortality and physical comorbidities of people with SMD and of risk factors and treatment strategies to improve the health and life of people with SMIs. All these issues have been addressed in the Research Topic. Six accepted papers are original research (4 are Original Research Papers and 2 are Brief Research Reports); moreover, the issue also includes 1 Review, 1 Policy and Practice Review paper, 1 Mini Review, 1 Perspective and 1 Case report paper, covering most aspects related to the management of physical comorbidity in patients with severe mental disorders.

Several papers included in this Research Topic delt with the clinical characterization of patients with severe mental disorders at risk of developing physical illnesses. The paper by Isella et al. focused on the role of resilience in a sample of patients who received an implantable cardioverter defibrillator; Sampogna et al. explored, in a sample of patients with SMD, the influence of recovery style on patients' engagement in healthy lifestyle behaviors, physical activity and improvement of dietary patterns. Baron et al. have addressed the issue of physical activity of patients with SMD, reporting that interventions associated with an improvement of physical activity levels can reduce patients' overall mortality. The paper by Berardelli et al. dealt with the relationship between lifestyle behaviors, mental health, and suicide risk and ideation.

The relationship among mental and physical health during emergencies have been addressed by De Hert et al. and by Medved et al. In particular, De Hert et al. provided an overview of published studies addressing the increased mortality rates of patients with SMD during the COVID-19 pandemic, while Medved et al. described the impact of the 2020 Croatian devastating earthquake on the physical and mental health of people with SMD Cuomo et al. focused on the inflammatory processes and on the association between inflammation and onset and maintenance of mental disorders showing an increase of a series of inflammatory markers in acute phase of patients with bipolar disorder, and the normalization of these indexes associated with the improvement of the patients' mental health status.

Four papers included in this Research Topic deal with interventions aiming at improving the physical health of people with severe mental disorders. Ventriglio et al. tested the efficacy of a psychosocial intervention providing information about the possible metabolic side effects of antipsychotics and their prevention and management. They reported that patients with SMD receiving the experimental intervention reported an improvement of BMI, a decrease in serum levels of fasting glucose, hemoglobin glycosylation and cholesterol, along with an improvement in mental health-related domains. The challenges in the implementation of psychosocial interventions in routine practice has been addressed by Yuan et al. and by Kohn et al. In particular, Yuan et al. reported results of the implementation of interventions related to (1) weight loss; (2) tobacco smoking cessation; and (3) hypertension, dyslipidaemia and diabetes care, in a sample of patients with SMD. Kohn et al., in their original research paper, analyzed patients' and healthcare professionals' perspectives on somatic health in three psychiatric settings. They reported that stigma, communication difficulties among professionals and organizational difficulties (i.e., low availability of equipment, reduced building capacity, understaffing) are the most important factors hindering the achievement of satisfying levels of physical health in people with severe mental disorders.

Lastly, in their Policy and Practice Review, Falkai et al. present in overview of the activity of the “Munich/Augsburg consortium Precision in Mental Health (PriMe),” which will develop a global research framework aiming to deepen the understanding of comorbidities of patients with SMD and to identify and validate predictive markers of chronicity and mortality in in routine settings The PriMe Consortium will also aim to develop novel multimodal treatments, identify strategies to disseminate personalized treatments and ways to test their effectiveness, utility and scalability.

Taken together, the papers included in this Research Topic provide new knowledge and insights about the comorbidity of mental and physical disorders; they also highlight that much more work needs to be done. Over the past 10 years research on the complex interplay between mental and physical health has rapidly progressed and produced important evidence In the years to come, research should focus on the identification of protective factors that could reduce comorbidity of mental and physical disorders and reduce the mortality of people with severe mental disorders and on the acquisition of results which will allow the assessment of the value and cost-effectiveness of psychosocial interventions in dealing with the problems of comorbidity.

## Author contributions

All authors listed have made a substantial, direct, and intellectual contribution to the work and approved it for publication.

## Conflict of interest

The authors declare that the research was conducted in the absence of any commercial or financial relationships that could be construed as a potential conflict of interest.

## Publisher's note

All claims expressed in this article are solely those of the authors and do not necessarily represent those of their affiliated organizations, or those of the publisher, the editors and the reviewers. Any product that may be evaluated in this article, or claim that may be made by its manufacturer, is not guaranteed or endorsed by the publisher.
